# ^18^ F-Fluorocholine PET-CT Outshines Sestamibi Scintigraphy in Detecting Parathyroid Adenomas in the Background of Hashimoto's Thyroiditis

**DOI:** 10.1055/s-0043-1774732

**Published:** 2023-12-04

**Authors:** Piyush Chandra, Dipayan Nandy, Samir Saini

**Affiliations:** 1 Department of Nuclear Medicine, Zydus Hospitals, Vadodara, Gujarat, India; 2 Department of General Surgery, Parul Institute of Medical Sciences and Research, Vadodara, Gujarat, India; 3 Sanidhya Clinic, Vadodara, Gujarat, India

**Keywords:** autoimmune, FCH, fluorocholine, Hashimoto's, PET, sestamibi, thyroiditis

## Abstract

Ultrasonography neck and dual-phase ^99m^ Tc-sestamibi (MIBI) scan are standard imaging techniques for the detection of parathyroid adenomas in primary hyperparathyroidism. However, in presence of coexistent thyroid disease or small size of adenomas, the accuracy of these imaging modalities is low and leads to delayed diagnosis. We here present a report of two patients with primary hyperparathyroidism and with a nondiagnostic MIBI scan, who subsequently underwent successful surgery after positive localization of adenomas on ^18^ F-fluorocholine positron emission tomography-computed tomography.

## Introduction

Coexisting thyroid diseases are not uncommon in patients undergoing surgery for primary hyperparathyroidism (PHPT). Incidence of Hashimoto's thyroiditis (HT) has been reported in 8 to 18% of patients with PHPT with slightly higher rate of prevalence of PHPT in patients with HT and resulting hypothyroidism than compared to the general population (1.89 vs. 0.3%). 1 2 3 The exact mechanism of such association remains unclear; however, an experimental study in a rat model proved that a hypothyroid state or increased thyroid-stimulating hormone (TSH) could be the reason for the development of parathyroid adenomas. 4 From a molecular imaging perspective, coexisting thyroid diseases are known to reduce the overall diagnostic accuracy of ^99m^ Tc-sestamibi (MIBI) scintigraphy in diagnosing parathyroid adenomas in PHPT causing delay in treatment and increased patient suffering. MIBI single photon emission computed tomography-computed tomography (SPECT-CT), four-dimensional CT (4D-CT), ultrasound-guided fine-need aspiration cytology, and ^18^ F-fluorocholine (FCH) positron emission tomography-CT (PET-CT) are alternative techniques for detecting these apparently occult adenomas.

### Case1

**Fig. 1:**
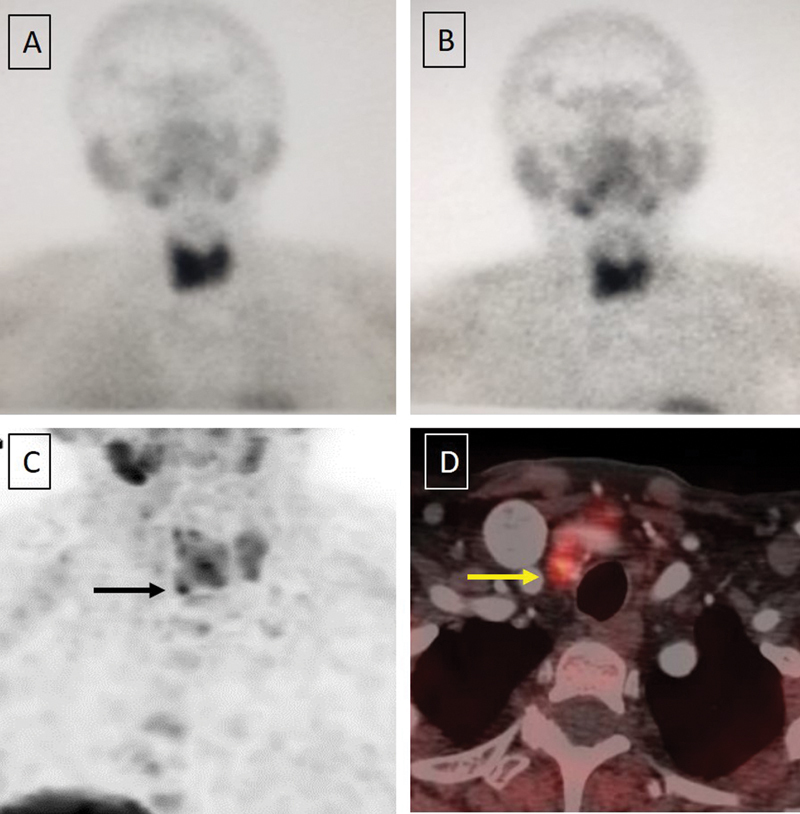
( **A** and **B** ) Sestamibi scan: Early 20 minutes ( **A** ) and delayed 120 minutes ( **B** ) planar sestamibi scan of the neck showing diffuse high-grade thyroid uptake with no focal uptake in the thyroid/parathyroid bed. ( **C** and **D** ) Fluorocholine (FCH) positron emission tomography-computed tomography (PET-CT), showing 60-minute static maximum intensity projection image showing low-grade uptake in both lobes of the thyroid with focal uptake noted in the right inferior pole of thyroid ( *black arrow* , **C** ) seen as an 8-mm nodule on fused PET-CT images ( *yellow arrow* , **D** ).

A 56-year-old lady presented with a history of generalized weakness and low backache. On biochemical evaluation, intact parathyroid hormone (iPTH) and serum calcium were elevated (133 pg/mL and 9.5 mg/dL). Antithyroid peroxidase 9.21 IU/mL (normal < 5.6), antithyroglobulin 380.50 IU/mL (normal < 115), and TSH 1.46 uIU/mL, suggestive of HT. Ultrasonography (USG) scan of the neck was reported as negative for parathyroid abnormality. MIBI scan showed diffuse increased uptake in both lobes of the thyroid with intense uptake in early ( Fig. 1A ) and no significant thyroid washout in delayed images ( Fig. 1B ). No focal retention in the thyroid bed was noted to suggest parathyroid adenoma. FCH PET-CT, done a week later, showed low-grade uptake in both lobes of the thyroid in early and delayed static images with focal uptake noted in the 8-mm nodule in the right inferior pole ( Fig. 1C,D , *black* and *yellow arrows* ) on 60-minute images (maximum standardized uptake value [SUVmax] 6.3), suggestive of parathyroid adenoma. Findings of FCH PET-CT were confirmed intraoperatively and adenoma was excised, intraoperative PTH declined from 73 to 13 pg/mL. On follow-up, patient reported significant improvement in symptoms and normalizing of blood values of calcium (8.9 mg/dL) and iPTH (49.7 pg/mL).

### Case 2

**Fig. 2:**
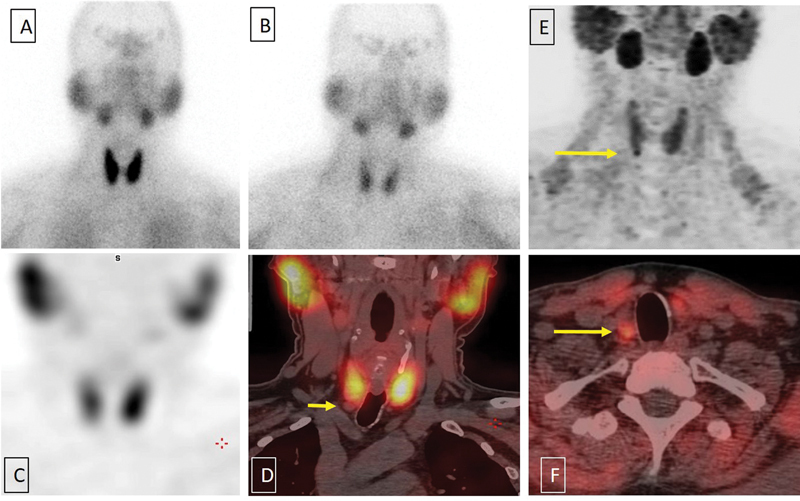
( **A** and **B** ) Sestamibi (MIBI) scan: Early 20 minutes planar sestamibi scan of the neck showing diffuse thyroid uptake with gradual washout on delayed 120-minute planar image with no focal uptake in the thyroid/parathyroid bed in early or delayed scans. ( **C** and **D** ) No focal uptake in thyroid bed on maximum intensity projection single photon emission computed tomography (SPECT) images ( **C** ) and non-MIBI avid 7.5-mm nodule noted in the right inferior pole of thyroid on fused SPECT-CT images acquired at 2 hours ( *short yellow arrow* , **D** ). ( **E** and **F** ) Fluorocholine (FCH) positron emission tomography-computed tomography (PET-CT), showing 60-minute maximum intensity projection image showing low-grade uptake in both lobes of the thyroid images with focal uptake noted in the right inferior pole of thyroid ( *long yellow arrow* , **E** ) seen as a 7.5-mm nodule on fused PET-CT images ( *long yellow arrow* , **F** ).

A 70-year-old male with a history of HT and primary hypothyroidism, on levothyroxine (12.5 µm/day), complained of generalized weakness and recurrent renal calculi for the past 2 years and was found to have elevated iPTH (195.2 pg/mL) and serum calcium (13.3 mg/dL) and high urinary calcium excretion of about 297 mg/day. TSH was 4.28 mIU/mL. USG scan of the neck was negative. MIBI scan was done which showed diffuse uptake in the thyroid in early images ( Fig. 2A ) with partial washout from the thyroid on delayed images with no focal uptake in either phase ( Fig. 2B ). No focal uptake in thyroid bed was seen on maximum intensity projection SPECT images ( Fig. 2C ) and non-MIBI avid 7.5-mm nodule noted in the right inferior pole of thyroid on fused SPECT-CT images acquired at 2 hours ( *short yellow arrow* , Fig. 2D ). FCH PET-CT was done a week later. Sixty-minute static PET images showed low-grade uptake in the thyroid with focal uptake in the 7.5-mm in the right inferior pole of the thyroid ( Fig. 2E,F , *long yellow arrows* , SUVmax 6.85), suggestive of parathyroid adenoma. Findings of FCH PET-CT were confirmed intraoperatively and a small cystic adenoma was excised with a decline in intraoperative iPTH levels. On follow-up, patient reported significant improvement in symptoms and normalizing of blood values of serum calcium (9.2 mg/dL) and iPTH (35.4 pg/mL). Reasons for discordance between SPECT-CT and PET-CT findings in this case could be related to lower spatial resolution of SPECT or rapid washout of MIBI from parathyroid tissue, as SPECT acquisition was done at 2 hours postinjection.

## Discussion

USG with MIBI scintigraphy are currently the standard imaging modalities for preoperative localization of parathyroid adenoma. Small size, low oxyphil cell content, cystic degeneration in parathyroid adenomas, and presence of autoimmune thyroid disease are some of the factors associated with false-negative MIBI scans for parathyroid adenoma detection. 5 6 7 In cases of hypertrophic phase of HT, the mean uptake of the MIBI in the thyroid and clearance T1/2 have shown to be significantly higher compared to those of euthyroid volunteers. 8 Not surprisingly, in the presence of HT and resulting higher background thyroid activity, small parathyroid adenomas are bound to be missed on MIBI scan acquired on gamma camera which have limited spatial resolution. Boi et al reported an 80% false-negative rate (FNR) for MIBI scans in patients with autoimmune thyroiditis, significantly higher compared to an FNR of 35% for patients with nodular goiter. 7

SPECT-CT, by providing both functional and anatomical correlation, has been shown to have higher diagnostic accuracy compared to planar scintigraphy for detecting and localizing parathyroid adenomas in patients with autoimmune thyroiditis. 9 In the study done by Hwang et al, per lesion sensitivity was higher for SPECT-CT compared to planar scans (80 vs. 57.8%, *p* = 0.02) in patients with MIBI retention in the thyroid. No significant difference was noted in sensitivity of the two methods in patients with good MIBI washout from the thyroid.

4D-CT is another established technique for the detection of parathyroid adenomas with pooled per patient sensitivity and specificity of 81 and 91%, respectively. 10 Typical parathyroid adenomas are hypoattenuating to thyroid on noncontrast phase with avid arterial enhancement and rapid washout on the venous phase. Disadvantages of 4D-CT are variable enhancement patterns (seen in up to one-third of adenomas), use of iodinated contrast (making it a nonviable option in patients with chronic kidney disease), and excess radiation exposure compared to existing techniques. 11 The effective radiation dose of 4D-CT scans (10.4–28 mSv) is reported to be higher than that with FCH PET-CT (6 mSv) and dual-phase MIBI scan (6.3 mSv). 12

Comparable to MIBI scans is FCH PET-CT which offers shorter imaging time, better spatial resolution, and a better lesion-to-background ratio, leading to higher detection rates. Exact mechanism of FCH in parathyroid adenomas is not completely understood. It could be related to high expression of choline kinase enzyme in the adenomas, which are responsible for phosphorylation of choline. 12 Recently published meta-analysis by Whitman et al (data from 20 studies including 796 patients from 2014 to 2020) showed a significantly better sensitivity for FCH PET (96%) compared to MIBI scan (54%) for parathyroid adenomas detection. 13 Zajíčková et al showed a parathyroid adenoma detection rate of 92% on FCH PET-CT in 12/13 patients with coexisting thyroid disease and inconclusive conventional imaging. 14 FCH PET can be combined with 4D contrast-enhanced CT, offering a complete “one-stop” diagnostic imaging solution for detecting and localizing parathyroid adenomas.

## Conclusion

FCH PET-CT can be considered an ideal first-line imaging modality for the detection of parathyroid adenomas in patients with coexisting HT, who are prone for a false-negative MIBI scan given the high MIBI retention in the thyroid.
